# A Systematic Review of the Incidence and Pattern of Surgical Site Infection in Orthopedic Surgery in Africa

**DOI:** 10.7759/cureus.71084

**Published:** 2024-10-08

**Authors:** Obinna E Ikegwuonu, Collins C Okeke, Abdulahi Zubair, Promise U Okereke, Somadila A Igboanugo, Emmanuel O Oladeji, Olaoluwa E Ebiekuraju

**Affiliations:** 1 Orthopaedics, Surgery Interest Group of Africa, Lagos, NGA; 2 Anaesthesiology, Surgery Interest Group of Africa, Lagos, NGA; 3 Internal Medicine, University of Port Harcourt, Port Harcourt, NGA; 4 General Surgery, Surgery Interest Group of Africa, Lagos, NGA; 5 Dentistry, University College Hospital, Ibadan, NGA; 6 Cardiology, Rostov State Medical University, Rostov-on-Don, RUS; 7 Trauma and Orthopaedics, Surgery Interest Group of Africa, Lagos, NGA

**Keywords:** africa, orthopedic procedures, orthopedic surgery, surgical site infection, wound infection

## Abstract

Surgical site infections (SSIs) are a major health challenge in Africa, leading to poor patient outcomes. This study aims to systematically review and summarize existing research on the rate and patterns of SSIs in orthopedic surgery across Africa. A thorough search was conducted using databases such as Embase (via Ovid), PubMed, Scopus, African Journals Online (AJOL), and Google Scholar for literature published between January 2000 and July 2024. The search used terms such as "surgical site infection," "SSI," "surgical wound infection," "orthopedic surgery," and "Africa." After screening, studies that did not meet the criteria were excluded, leaving seven studies (five retrospective and two prospective) with 989 patients who had undergone orthopedic procedures in both elective and emergency settings. The overall incidence of SSIs was 10.5%, affecting 104 patients, with rates ranging from 4.2% in Ethiopia to 39% in Togo. Of the infections reported, 43% were superficial, 26% were deep, 11% affected organ space, and 20% were chronic. This review sheds light on the high rates of SSIs in orthopedic surgeries in Africa, emphasizing the need for better infection control and improved surgical practices. More research is necessary to fill the gaps and develop strategies that can be applied in different healthcare settings.

## Introduction and background

Surgical site infections (SSIs) are a common complication following surgical procedures in humans. It is a dreaded complication of surgery, and identifying risk factors for patients can help manage their expectations and improve recovery outcomes. The Centers for Disease Control and Prevention (CDC) and the European Centre for Disease Prevention and Control (ECDC) define a surgical site infection (SSI) as an infection occurring within 30 days after a surgical procedure or one year if a permanent implant is involved [1-3]. The most common causative microorganisms reported are *Escherichia coli* and *Staphylococcus aureus* [4].

SSIs account for many nosocomial infections among surgical patients, ranking third (14.2%) among causes of infection in patients after urinary tract infections (36%) and respiratory infections (12%) [5]. SSIs are among the greatest burdens of healthcare-associated infections, especially in resource-limited regions such as Africa. The estimated prevalence is 15.5 per 100 patients in Africa, compared to 7.1 per 100 patients in Europe and 4.5 per 100 patients in the United States [6]. Several studies report high rates of SSIs in Sub-Saharan Africa, such as 8% in Kenya, 22% in a rural Tanzanian hospital, and 9.9% in Nigeria [7].

The Centers for Disease Control and Prevention (CDC) identify SSIs based on several criteria such as purulent drainage from a superficial incision, deep incision, or organ space, identification and culture of an organism from an aseptically obtained fluid or tissue sample, and an incision that spontaneously dehisces or is deliberately opened by a surgeon or physician, along with at least one of the following: localized pain, tenderness, erythema (heat, erythema, and swelling), or fever (>38°C) and evidence of an abscess on imaging or during surgical revision [8]. A similar pattern of surgical site infections is seen in orthopedics, with the estimated annual incidence following all orthopedic procedures between 31,000 and 35,000 [9]. The risk factors for surgical site infections in orthopedic surgery are similar to the overall risks and should be carefully considered for all patients. Patients over 60 are at increased risk due to reduced immunity with age [5,7]. Preoperative shaving also affects SSI risk as patients who do not have their hair shaved are at greater risk, and shaving with hand razors increases the risk more than using electric razors [5,8]. A longer hospital stay post-operation increases the risk of developing an SSI, likely due to skin colonization by nosocomial organisms that resist antibiotics used for preoperative prophylaxis [7]. The risk of SSI rises particularly in surgeries lasting over two hours, and several other factors such as increased wound contamination, the number of sutures, blood loss, and reduced effectiveness of prophylactic antibiotics also contribute.

Additionally, the use of implants is linked to a higher incidence of surgical site infections [7]. Systemic comorbidities such as poorly controlled diabetes mellitus and malnutrition are moderate risk factors for SSIs [8]. In orthopedic surgery, the risk of SSIs is higher due to the nature of these surgeries, the highly comorbid nature of the elderly demographics requiring complex surgeries, and the inherent higher infection risks associated with procedures such as arthroplasty or treating open injuries [4]. Approximately 14.4% of orthopedic surgery patients experience adverse effects during perioperative care, with infections being the most prevalent complication, leading to SSIs in 1.3% of cases [10]. The rising prevalence of orthopedic surgeries has led to an increase in SSIs within this field. SSIs are classified as superficial incisional (those involving only the skin or subcutaneous tissue), deep incisional (those involving deep soft tissues of an incision), and organ space (those involving organs or body spaces). Globally, several factors are associated with orthopedic SSIs, including low serum albumin levels (serum albumin < 36.7-41.6 g/L), high body mass index (BMI) (BMI > 28 kg/m^2^), smoking, open fractures, multiple fractures, contaminated wounds, the presence of surgical drains, diabetes, and surgeries in areas with deep-seated infections. Other contributing factors include the level of the surgeon's experience, reoperation, prolonged surgery duration, male sex, and extended postoperative hospital stay [10].

SSIs caused by *Staphylococcus aureus* colonization are more common in patients undergoing total joint arthroplasty than in other orthopedic procedures, accounting for over 60% of infections complicating total hip or knee arthroplasty. The cumulative incidence of periprosthetic joint infection after primary total hip or knee arthroplasty is 1.4% after 10 years, with a five-year mortality rate of 21.12% [11]. In orthopedic studies, SSIs following osteosynthesis of trochanteric and subtrochanteric fractures occur in approximately 1%-3% of patients [12]. Post-orthopedic surgery SSIs impose a high burden of higher mortality, increased hospital stay, and greater costs to the healthcare system [12,13]. Considering the significant burden of surgical site infections (SSIs) and their impact on surgical outcomes, it is crucial to adhere to the CDC guidelines for preventing SSIs based on a modified Grading of Recommendations, Assessment, Development, and Evaluation (GRADE) approach [14].

Currently, there is a limited body of published evidence looking at the pattern of SSIs in orthopedic practice in Africa, and population-based studies on their incidence are lacking. Addressing this research gap in this systematic review, we aim to examine the incidence and patterns of surgical site infection in orthopedic practice from African centers.

## Review

Methods

This systematic review was conducted in accordance with the Preferred Reporting Items for Systematic Reviews and Meta-Analyses (PRISMA) guidelines [15]. This systematic review is registered with the International Prospective Register of Systematic Reviews (CRD42024577280).

Inclusion Criteria

All retrieved publications were included if they were primary studies conducted between January 2000 and July 2024, were published in English, and appeared in peer-reviewed journals. The studies had to report on the incidence and patterns of surgical site infection (SSI) among patients of any age and gender who underwent orthopedic procedures in healthcare facilities across Africa.

Exclusion Criteria

Studies involving non-human subjects, conducted outside Africa, or unavailable in full-text format were excluded. Additionally, case reports, audits, opinions, reviews, meta-analyses, comments, and editorials were not included.

Search Strategy

Two investigators (OEI and CCO) employed a comprehensive search strategy across PubMed, Embase, Scopus, African Journals Online (AJOL), and Google Scholar. Search terms included "surgical site infection," "surgical wound infection," or "SSI" in conjunction with "orthopaedic surgery," "orthopaedic procedure," or "orthopaedic," and "Africa." Studies carried out from January 2000 were candidates for inclusion. Search terms and details are provided in Table 1.

**Table 1:** Search strategy AJOL: African Journals Online, MeSH: Medical Subject Headings

Search	Database	Papers
1. Surgical site infection.mp. or exp surgical infection/ 2. orthopaedic surgery.mp. or exp orthopedic surgery/ 3. exp Africa/	Embase (Ovid)	80
(orthopaedic surgical site infection) AND (Africa)	PubMed	105
((Surgical Wound infection) AND (Orthopaedic)) AND (Africa)	PubMed	84
(("Surgical Wound Infection"[MeSH]) AND "Orthopedics"[MeSH]) AND "Africa"[MeSH]	PubMed	6
(((surgical site infection) OR (SSI)) AND (Orthopaedic surgery)) AND (Africa)	PubMed	80
"orthopaedic" "surgical site infection" "Africa"	Scopus	12
"orthopaedic" "surgical wound infection"	Scopus	9
"orthopaedic" "surgical site infection" "Africa"	AJOL	48
"orthopaedic" "surgical site infection" "Africa"	Google Scholar	The first 100 pages were searched; 21 studies were identified.

Study Selection

Two independent reviewers (OEI and CCO) screened titles and abstracts and subsequently assessed full-text articles. Disagreements were resolved by discussion or consultation with a third reviewer.

Data Extraction and Synthesis

Data was extracted by two independent authors and charted using a Microsoft Office Excel version 16 (Microsoft Corp., Redmond, WA) proforma. Specifically, they extracted data from articles related to the study characteristics (author, year, country, and setting), patient demographics (age and sex), type of orthopedic surgery, incidence rate of surgical site infection (SSI), patterns of SSI (e.g., superficial, deep, and organ/space), risk factors associated with SSI, cultured organisms, outcomes (morbidity and mortality), timing of SSI, and method of diagnosis.

The methodological quality of the included studies was assessed using the Newcastle-Ottawa Scale (NOS) [16]. In the Newcastle-Ottawa Scale (NOS), stars are assigned in three categories: selection, which can receive between 0 and 3 stars; comparability, which ranges from 0 to 2 stars; and outcome for cohort studies or exposure for case-control studies, with a possible 0 to 3 stars. Studies that received a total of seven or more stars on the NOS were considered to be of high quality. The results of the risk of bias assessment are provided in Table 2 and Table 3 [17-23].

**Table 2:** Newcastle-Ottawa Scale (cohort study)

Study	Selection				Comparability	Outcome			Total
	Representativeness of the exposed cohort	Selection of the non-exposed cohort	Ascertainment of exposure	Outcome not present at the start of the study		Assessment of outcome	Length of follow-up	Adequacy of follow-up	
Bossa et al. [17]	*		*	*	**	*	*	*	********
Abalo et al. [18]	*		*	*	**	*	*	*	********
Young et al. [20]	*		*	*	**	*	*	*	********
Akinyoola et al. [21]	*		*	*	**	*	*	*	********
Ekwunife et al. [23]	*		*	*	**	*	*	*	********

**Table 3:** Newcastle-Ottawa Scale (case-control study)

Study	Selection				Comparability	Exposure			Total
	Adequacy of case definition	Representativeness of case	Selection of control	Definition of control		Ascertainment of exposure	Same method of ascertainment (cases and control)	Non-response rate	
Beza et al. [19]	*	*	*	*	**	*	*		********
Ojo et al. [22]	*	*	*	*	**	*	*		********

Result

**Figure 1:**
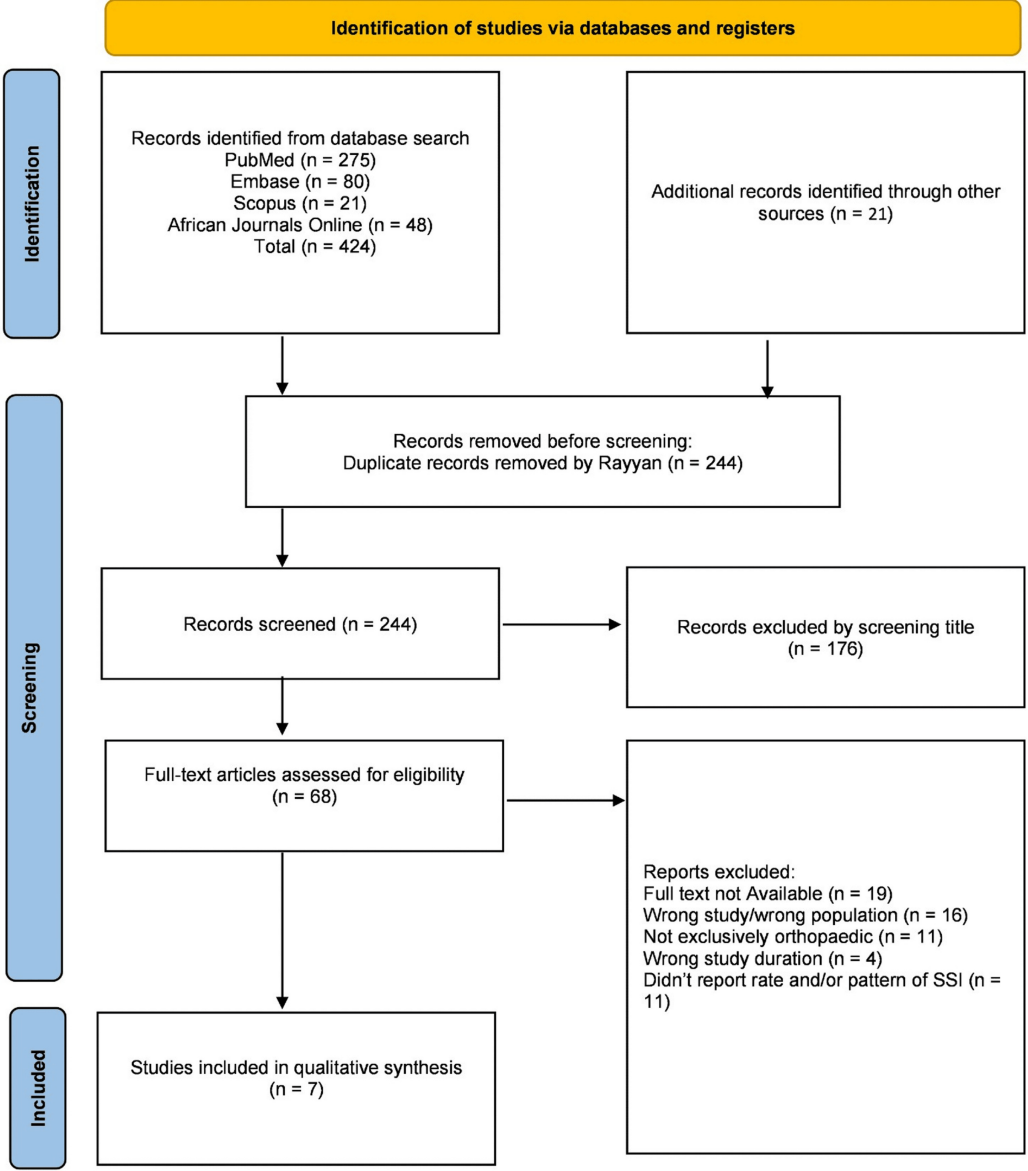
PRISMA 2020 flow diagram for new systematic reviews incorporating database and register searches only PRISMA: Preferred Reporting Items for Systematic Reviews and Meta-Analyses

A systematic search of the databases identified 445 studies, as shown in Figure 1. After removing duplicates, 244 studies remained, of which 176 were excluded based on abstract screening. Following a full-text review of the remaining 68 papers, only seven studies fully met the eligibility criteria according to the previously mentioned inclusion and exclusion guidelines.

The studies included spanned a period of 24 years, from 2000 to 2024. The studies included are classified as retrospective, retrospective cross-sectional, or prospective and are presented in Table 4.

**Table 4:** Study design of the included articles HIV: human immunodeficiency virus

Author	Study title	Type of study	Year of study	Country of study
Bossa et al. [17]	Surgical site infection in orthopedic and trauma surgery at Kinshasa University clinics	Retrospective cohort study	2010-2021	Congo
Abalo et al. [18]	Risk factors for surgical wound infection in HIV-positive patients undergoing surgery for orthopaedic trauma	Retrospective study	2005-2007	Togo
Beza et al. [19]	Infection after surgical implant generation network (SIGN) nailing in treatment of long bone shaft fractures in Ethiopia: analysis of a 4-year results	Retrospective study	2015-2018	Ethiopia
Young et al. [20]	Complications after intramedullary nailing of femoral fractures in a low-income country	Prospective study	2010-2012	Malawi
Akinyoola et al. [21]	Factors influencing the outcome of elective paediatric orthopaedic operations in Ile-Ife, Nigeria	Retrospective study	2000-2005	Nigeria
Ojo et al. [22]	Surgical site infection in posterior spine surgery	Retrospective cross-sectional study	2012-2014	Nigeria
Ekwunife et al. [23]	Comparative prospective study of early outcomes after osteosynthesis with locked intramedullary nailing or plating for closed femoral shaft fractures at the National Orthopaedic Hospital Enugu, Nigeria	Prospective study	2015-2016	Nigeria

Sociodemographic Characteristics

Three of the seven eligible studies were conducted in Nigeria [21-23]; other countries include Malawi, Togo, Congo, and Ethiopia [17-20]. A total of 989 patients who underwent orthopedic surgery participated in the studies. The average age of participants was 31.8 ± 12.6 years with an age range of <1-85 years, and about 70.07% of the subjects were male.

Incidence of Surgical Site Infection and Other Clinical Variables

In this review, surgical site infections (SSIs) were documented in 104 out of 989 patients who underwent orthopedic procedures, resulting in an overall incidence rate of 10.5%. This incidence was calculated by dividing the number of patients who developed SSIs (104) by the total number of patients included in the studies (989) and multiplying by 100 to express the result as a percentage. However, it ranges from as low as 4.2% in Ethiopia to as high as 39% in Togo. This high rate of surgical site infection in Togo was reported among human immunodeficiency virus (HIV)-positive patients who underwent surgical fixation of fractures. The 10.5% rate provides a clear picture of the prevalence of SSIs in orthopedic surgeries across different African settings.

Factors Associated With Surgical Site Infection

Several comorbidities have been identified in the studies as contributing factors to the development of surgical site infections (SSIs). Human immunodeficiency virus (HIV) is a common condition linked to increased susceptibility to infections due to its impact on the immune system. Diabetes mellitus, with its associated complications such as poor wound healing and impaired immune responses, was also frequently noted. Tuberculosis (TB), another prevalent comorbidity, may contribute to SSIs through its chronic nature and the overall weakened state it causes in affected individuals. Obesity emerged as a risk factor as well, given the challenges it presents in wound healing, increased tissue stress, and compromised blood circulation. Anemia, which impairs oxygen delivery to tissues and may delay healing, was also recognized, along with hypertension, which can lead to vascular complications, further increasing the risk of infections post-surgery. Together, these conditions highlight the complexity of managing surgical outcomes in patients with multiple underlying health issues.

Surgical Procedures

Different orthopedic procedures were performed electively or in an emergency setting. These include hip hemiarthroplasty, internal and external fixation of fractures, corrective osteotomies, triple arthrodesis, excision of Baker's cyst, and spine surgeries. The duration of surgery was provided in all but three of the studies reviewed [17,20-22], with the duration ranging between less than one hour and greater than four hours. Prophylactic antibiotics were given in five of the studies reviewed [18-22], with intravenous cephalosporins being the most used class of antibiotics [19,20].

Surgical Site Infections

Regarding the number of surgical site infections (SSIs) recorded, a total of 104 cases were recorded across all the studies reviewed as presented in Table 5.

**Table 5:** Number and incidence of SSI in each study SSI: surgical site infection, HIV: human immunodeficiency virus

Author	Study title	Number of SSIs	SSI rate (%)
Bossa et al. [17]	Surgical site infection in orthopaedic and trauma surgery at Kinshasa University clinics	37	15
Abalo et al. [18]	Risk factors for surgical wound infection in HIV-positive patients undergoing surgery for orthopaedic trauma	14	39
Beza et al. [19]	Infection after surgical implant generation network (SIGN) nailing in treatment of long bone shaft fractures in Ethiopia: analysis of a 4-year results	13	4.20
Young et al. [20]	Complications after intramedullary nailing of femoral fractures in a low-income country	8	6
Akinyoola et al. [21]	Factors influencing the outcome of elective paediatric orthopaedic operations in Ile-Ife, Nigeria	17	8.10
Ojo et al. [22]	Surgical site infection in posterior spine surgery	10	16.10
Ekwunife et al. [23]	Comparative prospective study of early outcomes after osteosynthesis with locked intramedullary nailing or plating for closed femoral shaft fractures at the National Orthopaedic Hospital Enugu, Nigeria	5	9.6

When classifying surgical site infections (SSIs), they are grouped as either superficial, deep, or organ/space infections based on their characteristics. A total of 40 instances of superficial infections and 24 deep infections were recorded, accounting for 43% and 26% of the reported infections, respectively. Additionally, organ/space infections represented 11% of the total infections. None of the studies provided the incidence of acute infections, but chronic infections (20%) were reported in the reviewed studies. The timing of SSI detection was presented in two studies, and it varied from five days post-operation to 17 months post-operation, with the average detection time being five days according to studies [20,22].

The primary pathogens were documented in three studies [17,22,23], identified as *Staphylococcus aureus*, *Pseudomonas* spp., and *Enterococcus* spp. However, the review did not include details on the sensitivity patterns or frequency of the cultures for these organisms.

Identified Risk Factors

Table 6 itemizes the given risk factors for SSIs from the studies included in this review.

**Table 6:** Risk factors for SSIs SSIs: surgical site infections, N/A: not available, HIV: human immunodeficiency virus

Author	Study title	Identified risk factors	Method of diagnosing SSIs
Bossa et al. [17]	Surgical site infection in orthopaedic and trauma surgery at Kinshasa University clinics	Male gender, emergency surgery, preoperative duration greater than 24 hours	N/A
Abalo et al. [18]	Risk factors for surgical wound infection in HIV-positive patients undergoing surgery for orthopaedic trauma	HIV status, CD4+ T-lymphocyte level, contaminated wounds (preoperative)	Clinical and microbiological
Beza et al. [19]	Infection after surgical implant generation network (SIGN) nailing in treatment of long bone shaft fractures in Ethiopia: analysis of a 4-year results	Mechanism of injury	N/A
Young et al. [20]	Complications after intramedullary nailing of femoral fractures in a low-income country	N/A	Clinical
Akinyoola et al. [21]	Factors influencing the outcome of elective paediatric orthopaedic operations in Ile-Ife, Nigeria	Preoperative diagnosis, length of operation, intraoperative blood loss above 200 mL	Clinical
Ojo et al. [22]	Surgical site infection in posterior spine surgery	Spinal instrumentation, surgery on cervical region, wound inspection on or before postoperative day 5, comorbidities	Clinical and microbiological
Ekwunife et al. [23]	Comparative prospective study of early outcomes after osteosynthesis with locked intramedullary nailing or plating for closed femoral shaft fractures at the National Orthopaedic Hospital Enugu, Nigeria	N/A	Clinical and microbiological

Morbidity and mortality recorded included chronic osteomyelitis and non-union in the study of Abalo et al. [18], three mortalities in the study of Young et al. [20], and wound infection anemia in the study of Akinyoola et al. [21]. The mean follow-up period was 21.5 weeks [19] and 381 days [20].

Discussion

The results of this systematic review reinforce the existing literature on the significant burden of surgical site infections (SSIs) in orthopedic surgery, particularly in resource-limited settings such as Africa. The overall incidence of 10.5% found in this review aligns with other studies conducted in low- and middle-income countries, where SSIs are often more prevalent due to factors such as limited access to advanced surgical technologies, inconsistent implementation of infection control measures, and challenges in maintaining sterile environments [24]. The wide variation in surgical site infection (SSI) rates across different studies also mirrors findings from global research, highlighting how local factors such as patient comorbidities, healthcare infrastructure, and surgical practices influence infection outcomes [25]. These results underscore the urgent need for context-specific strategies to mitigate the risk of SSIs in these regions.

The review's inclusion of studies from multiple African countries over a 24-year period provides a comprehensive overview of the SSI burden in orthopedic surgery across diverse settings from the contemporary standpoint, which enables us to understand the pattern over the years. Also, the inclusion of both retrospective and prospective studies allows for a broad examination of SSIs, encompassing different methodological approaches and data collection periods. Furthermore, the use of a comprehensive risk of bias assessment tool improves the credibility of the study as it reduces the bias within the study.

Despite the strengths of this review, several limitations must be acknowledged. The included studies exhibited considerable heterogeneity in terms of study design, patient populations, and surgical procedures. This variability complicates the direct comparison of results across studies and may limit the generalizability of the findings. Additionally, the absence of detailed microbiological data in some studies, particularly regarding pathogen resistance patterns, represents a significant gap in the literature. Understanding the local microbiological landscape is critical for guiding appropriate antibiotic prophylaxis and treatment strategies.

Incidence of SSI in Orthopedic Surgery in Africa

The review revealed a substantial range in SSI rates, from as low as 4.20% in Ethiopia [19] to as high as 39% in Togo [18]. This variability is indicative of the diverse healthcare contexts across Africa, where differences in surgical practices, patient demographics, and healthcare infrastructure can significantly impact infection rates [26]. The overall SSI rate of 10.5% is concerning when compared to global benchmarks, which typically report lower rates in more developed healthcare settings [27]. This elevated rate in African contexts could be attributed to a combination of factors including inadequate sterilization practices, limited access to prophylactic antibiotics, and the prevalence of high-risk patient populations, such as those with HIV, who are more susceptible to infections. The identification of these risk factors provides actionable insights for clinicians aiming to reduce the incidence of SSIs through targeted preoperative assessment and intraoperative management strategies [28].

Pattern of SSI in Orthopedic Surgery in Africa

The patterns of SSIs observed across the studies showed a mix of superficial and deep infections, with superficial infections being slightly more common (43% of reported infections) than deep infections (26%). The rate of superficial and deep infections is noticeably higher compared to what was reported in a study conducted in Malaysia [29], which found rates of 25% and 6.25% respectively. However, the overall trend is still quite similar. The deep/organ/space infection rate of 11% observed in this review is significantly lower compared to the findings from a study conducted in Portugal [30], which reported a notably higher infection rate of 64.5%. The discrepancy between the two studies could be attributed to several factors, such as differences in patient populations, types of orthopedic surgeries performed, infection surveillance methods, or implementation of preventive measures such as antibiotic prophylaxis and advanced wound care. The diversity in infection types underscores the complexity of SSIs in orthopedic surgery, where the type of surgery and the patient's underlying health status can influence the depth and severity of infections [31]. This pattern is consistent with other studies that have shown a high incidence of deep infections in orthopedic surgeries, often leading to more severe complications such as osteomyelitis and prolonged hospital stays [32].

Implicated Organisms

The review identified *Staphylococcus aureus*, *Pseudomonas* spp., and *Enterococcus* spp. as the main pathogens responsible for SSIs in the reviewed studies. These organisms are consistent with global findings, where *Staphylococcus aureus* is frequently reported as the most common causative agent in SSIs due to its ability to colonize the skin and surgical wounds [33]. The presence of *Pseudomonas* spp. and *Enterococcus* spp. also reflects the complex microbiological environment of SSIs in orthopedic surgery, where biofilm formation and antibiotic resistance can complicate treatment and lead to persistent infections [34].

Causes of SSI in Orthopedic Surgery in Africa

Several risk factors for SSIs were identified in the studies, including comorbid conditions such as HIV, diabetes mellitus, tuberculosis, and hypertension. Surgical factors such as the length of the procedure, the type of surgery performed, and the timing of prophylactic antibiotics were also significant contributors to the development of SSIs [18,19,21,22], which is consistent with previous findings in a more developed setting [3]. These findings are in line with global evidence that highlights the multifactorial nature of SSIs, where both patient-related factors and intraoperative variables play crucial roles [24]. The high incidence of SSIs among HIV-positive patients in Togo, for example, underscores the need for targeted interventions in high-risk populations [26].

Implications for Practice, Policy, and Future Research

The findings emphasize the need for improved infection control practices in orthopedic surgery across Africa, including rigorous sterilization protocols, appropriate use of prophylactic antibiotics, and closer monitoring of high-risk patients, particularly those with comorbid conditions [35].

Policymakers should prioritize the development of national guidelines for the prevention and management of SSIs, tailored to the specific healthcare environments in African countries. Investments in training healthcare providers in infection control and providing necessary resources are critical for reducing SSI rates.

There is a need for larger, multicenter prospective studies that can provide more definitive data on the incidence, risk factors, and outcomes of SSIs in orthopedic surgery across Africa. Research should also focus on understanding the microbiological landscape, including antibiotic resistance patterns, to inform more effective treatment strategies. Additionally, exploring the long-term outcomes of SSIs, such as their impact on patient quality of life and healthcare costs, will be essential for developing comprehensive interventions.

Strengths and Limitations of the Study

This systematic review of surgical site infections (SSIs) in orthopedic surgery across Africa has notable strengths and limitations that influence the reliability and applicability of its findings. Acknowledging these factors is crucial for interpreting the results within the context of healthcare in low- and middle-income countries (LMICs) and guiding future research.

A key strength of this review is its geographical diversity, incorporating studies from various African countries (e.g., Nigeria, Togo, Ethiopia, Congo, and Malawi). This broad perspective on SSI burden is valuable because healthcare environments, surgical practices, and infection risk factors can vary significantly across regions. By including multiple countries, the review offers a more comprehensive understanding of SSIs in African orthopedic surgery. Additionally, the review spans studies conducted between 2000 and 2024, allowing for an analysis of how SSI rates and related factors have changed over time, which is essential for identifying trends in infection control and healthcare improvements.

Another strength is the inclusion of diverse study designs: retrospective, retrospective cross-sectional, and prospective studies. This methodological variety enriches the data, as retrospective studies provide historical insights, while prospective studies reflect current practices and outcomes. This combination helps mitigate the limitations of relying solely on historical or current data, leading to a more nuanced understanding of SSI influencing factors.

However, the review also has limitations that affect its conclusions. One major limitation is the heterogeneity among the included studies. The wide variation in study designs, patient populations, and surgical procedures complicates direct comparisons and consistent conclusions. For example, some studies targeted patients with specific comorbidities, such as HIV, while others included a broader patient base. Additionally, the range of orthopedic surgeries varied from simple to complex procedures, limiting the generalizability of the findings, as SSI risk factors may differ significantly based on surgery type, patient health, and local practices.

Another limitation is the incomplete data in some studies. While the review identified key pathogens associated with SSIs (e.g., *Staphylococcus aureus*, *Pseudomonas* spp., and *Enterococcus* spp.), several studies lacked detailed microbiological data, including antibiotic resistance patterns. This gap is critical for developing effective antibiotic prophylaxis and treatment protocols, limiting the ability to recommend targeted infection control strategies based on specific pathogens and resistance patterns in different settings.

The small number of studies included in the final analysis, seven out of an initial 445 identified, also poses a limitation. Although the studies were selected based on rigorous criteria, the small sample size reduces the review's statistical power, making it challenging to draw definitive conclusions about surgical site infection (SSI) prevalence across Africa or to identify universal risk factors. Furthermore, the predominance of retrospective studies limits the ability to establish causality or control for confounding variables affecting SSI rates. Lastly, The variability in diagnosing and reporting surgical site infections (SSIs), combined with differences in follow-up periods, hinders the consistency of findings.

## Conclusions

This systematic review offers valuable insights into the epidemiology of surgical site infections (SSIs) in orthopedic surgery across several African countries. The findings emphasize the significant burden of SSIs in resource-limited settings and highlight the urgent need for continued efforts to improve surgical care and infection prevention practices. Tailored interventions are crucial, including the development of standardized infection prevention protocols and surgical preparation guidelines and the rational use of antibiotics both pre- and postoperatively. Additionally, patient education, infection control monitoring, and accountability mechanisms are necessary components of an effective strategy. Basic infrastructure improvements, such as ensuring that sterilization equipment is both accessible and functional, along with local data collection and surveillance systems, are essential for success. Strengthening preoperative screening, optimizing patients' health before surgery, and routinely training surgical teams to minimize unnecessary delays are equally important. Establishing regular audit cycles focused on critical infection prevention measures is key to addressing the specific risk factors and challenges identified in this review. These steps are essential for reducing the incidence of SSIs and improving patient outcomes in resource-limited environments. Finally, further research is needed to close the gaps identified and to develop evidence-based strategies that can be implemented across diverse healthcare settings.

## References

[1] (2013). European Centre for Disease Prevention and Control: Surveillance of surgical site infections in Europe 2010-2011. https://www.ecdc.europa.eu/en/publications-data/surveillance-surgical-site-infections-europe-2010-2011.

[2] Culver DH, Horan TC, Gaynes RP (1991). Surgical wound infection rates by wound class, operative procedure, and patient risk index. Am J Med.

[3] Horan TC, Gaynes RP, Martone WJ, Jarvis WR, Emori TG (1992). CDC definitions of nosocomial surgical site infections, 1992: a modification of CDC definitions of surgical wound infections. Infect Control Hosp Epidemiol.

[4] Taherpour N, Mehrabi Y, Seifi A, Eshrati B, Hashemi Nazari SS (2021). Epidemiologic characteristics of orthopedic surgical site infections and under-reporting estimation of registries using capture-recapture analysis. BMC Infect Dis.

[5] Dégbey C, Kpozehouen A, Coulibaly D, Chigblo P, Avakoudjo J, Ouendo EM, Hans-Moevi A (2021). Prevalence and factors associated with surgical site infections in the university clinics of traumatology and urology of the National University Hospital Centre Hubert Koutoukou Maga in Cotonou. Front Public Health.

[6] Murphy RA, Okoli O, Essien I (2016). Multidrug-resistant surgical site infections in a humanitarian surgery project. Epidemiol Infect.

[7] Ikeanyi UO, Chukwuka CN, Chukwuanukwu TO (2013). Risk factors for surgical site infections following clean orthopaedic operations. Niger J Clin Pract.

[8] Hijas-Gómez AI, Lucas WC, Checa-García A (2018). Surgical site infection incidence and risk factors in knee arthroplasty: a 9-year prospective cohort study at a university teaching hospital in Spain. Am J Infect Control.

[9] Greene LR (2012). Guide to the elimination of orthopedic surgery surgical site infections: an executive summary of the Association for Professionals in Infection Control and Epidemiology elimination guide. Am J Infect Control.

[10] Salia SM, Amesiya R, Adedia D (2024). Prevalence and determinants of orthopedic surgical site infections in rural northern Ghana: a retrospective cohort study. Discov Public Health.

[11] Pietrzak JR, Maharaj Z, Mokete L (2020). Prevalence of Staphylococcus aureus colonization in patients for total joint arthroplasty in South Africa. J Orthop Surg Res.

[12] Sator T, Binder H, Payr S (2024). Surgical site infection after trochanteric and subtrochanteric fractures: a single centre retrospective analysis. Sci Rep.

[13] Hon YG, Demant D, Travaglia J (2023). A systematic review of cost and well-being in hip and knee replacements surgical site infections. Int Wound J.

[14] Borchardt RA, Tzizik D (2018). Update on surgical site infections: the new CDC guidelines. JAAPA.

[15] Page MJ, McKenzie JE, Bossuyt PM (2021). The PRISMA 2020 statement: an updated guideline for reporting systematic reviews. BMJ.

[16] Wells GA, Shea B, O'Connell D, Peterson J, Welch V, Losos M, Tugwell P (2024). The Newcastle-Ottawa Scale (NOS) for assessing the quality of nonrandomised studies in meta-analyses. https://www.ohri.ca/programs/clinical_epidemiology/oxford.asp.

[17] Bossa JE, Madee RB, Natuhoyila Nkodila A, Mbuyi MA, Mokassa LB (2024). Surgical site infection in orthopedic and trauma surgery at Kinshasa University clinics. J Ortho Sports Med.

[18] Abalo A, Patassi A, James YE, Walla A, Sangare A, Dossim A (2010). Risk factors for surgical wound infection in HIV-positive patients undergoing surgery for orthopaedic trauma. J Orthop Surg (Hong Kong).

[19] Beza B, Bitew A, Melesse DY (2022). Infection after surgical implant generation network (SIGN) nailing in treatment of long bone shaft fractures in Ethiopia: analysis of a 4-year results. Eur J Orthop Surg Traumatol.

[20] Young S, Banza LN, Hallan G (2013). Complications after intramedullary nailing of femoral fractures in a low-income country. Acta Orthop.

[21] Akinyoola AL, Adegbehingbe OO, Ogundele OJ (2008). Factors influencing the outcome of elective paediatric orthopaedic operations in Ile-Ife, Nigeria. Tanzan J Health Res.

[22] Ojo OA, Owolabi BS, Oseni AW, Kanu OO, Bankole OB (2016). Surgical site infection in posterior spine surgery. Niger J Clin Pract.

[23] Ekwunife RT, Iyidobi EC, Enweani UN, Nwadinigwe CU, Okwesili CI, Ekwedigwe HC, Obande BO (2022). Comparative prospective study of early outcomes after osteosynthesis with locked intramedullary nailing or plating for closed femoral shaft fractures at the National Orthopaedic Hospital Enugu, Nigeria. Int Orthop.

[24] Allegranzi B, Bagheri Nejad S, Combescure C (2011). Burden of endemic health-care-associated infection in developing countries: systematic review and meta-analysis. Lancet.

[25] Meara JG, Leather AJ, Hagander L (2016). Global Surgery 2030: evidence and solutions for achieving health, welfare, and economic development. Int J Obstet Anesth.

[26] Mawalla B, Mshana SE, Chalya PL, Imirzalioglu C, Mahalu W (2011). Predictors of surgical site infections among patients undergoing major surgery at Bugando Medical Centre in Northwestern Tanzania. BMC Surg.

[27] Langvatn H (2020). Infected total hip arthroplasty - bacteriology and the role of operating room ventilation in the reduction of postoperative infection. https://hdl.handle.net/1956/24102.

[28] Whitehouse JD, Friedman ND, Kirkland KB, Richardson WJ, Sexton DJ (2002). The impact of surgical-site infections following orthopedic surgery at a community hospital and a university hospital: adverse quality of life, excess length of stay, and extra cost. Infect Control Hosp Epidemiol.

[29] Chua W, Rahman S, Deris Z (2022). Prevalence, risk factors and microbiological profile of orthopaedic surgical site infection in north-eastern Peninsular Malaysia. Malays Orthop J.

[30] Guedes M, Almeida F, Andrade P, Moreira L, Pedrosa A, Azevedo A, Rocha-Pereira N (2024). Surgical site infection surveillance in knee and hip arthroplasty: optimizing an algorithm to detect high-risk patients based on electronic health records. Antimicrob Resist Infect Control.

[31] Kirkland KB, Briggs JP, Trivette SL, Wilkinson WE, Sexton DJ (1999). The impact of surgical-site infections in the 1990s: attributable mortality, excess length of hospitalization, and extra costs. Infect Control Hosp Epidemiol.

[32] Owens CD, Stoessel K (2008). Surgical site infections: epidemiology, microbiology and prevention. J Hosp Infect.

[33] Weigelt JA, Lipsky BA, Tabak YP, Derby KG, Kim M, Gupta V (2010). Surgical site infections: causative pathogens and associated outcomes. Am J Infect Control.

[34] Darouiche RO (2004). Treatment of infections associated with surgical implants. N Engl J Med.

[35] Leaper DJ, Edmiston CE (2017). World Health Organization: global guidelines for the prevention of surgical site infection. J Hosp Infect.

